# A new psycho-education module for adolescents with a first episode of psychosis and their relatives

**DOI:** 10.1192/j.eurpsy.2025.1623

**Published:** 2025-08-26

**Authors:** M. G. Pijnenborg, N. Boonstra, S. Sanches, D. Boertien, B. Stavenuiter, B. S. Rosema, C. Elzinga

**Affiliations:** 1 university of Groningen, groningen; 2 university of Utrecht; 3 KCPhrenos; 4 mind Ypsilon, Utrecht; 5 NHL Stenden, Leeuwarden, Netherlands

## Abstract

**Introduction:**

Psychotic disorders typically have their onset in adolescence, a formative age that comes with many developmental challenges. The onset of mental illness in this phase of life can be very disruptive. Literacy on the nature and potential impact of psychosis can promote recovery, while family interventions have the potential to prevent relapse. Moreover, many patients and family members report a need for tools that provide guidelines to improve their communication and stimulate mutual understanding. However, an integrated psyche-education module for both adolescents with lived experience and their relatives was lacking until now.

**Objectives:**

The development of a new integrated psycho-education module and the content of this module will be presented,

**Methods:**

Stakeholders (people with lived experience and family members) were interviewed on their preferences for the content, form and timing of psycho-education individually (n=15) and in focus groups (n=7). This information was used by a task force of experts composed of people with lived experience with psychosis, family members and mental health professionals. Subsequently, the intervention was piloted in two mental health care organizations.

**Results:**

Adolescents with lived experience preferred psycho-education on communications skills and wanted to learn how to communicate with their relatives during and after the psychotic episode, adjusted to their specific situation. They prefer psycho-education together with their relatives, not separately. They also noted that the content of psycho-education may differ depending on the relationship they have with their relatives. Relatives and people with lived experience both reported they needed basic knowledge about psychosis, training in communication and problem solving skills, content with respect to self-care and content about online information. With regards to the timing of psycho-education, relatives preferred basic information as soon as possible and communication skills later on.

Both groups felt an integrated psycho-education could be beneficial. The final integrated module is composed of themes related to different dimensions of recovery, e.g. societal and social impact and spirituality. Results of the pilot were used to refine the module.

**Conclusions:**

This new integrated psycho-education module was based on stakeholders preferences and needs and was found to be helpful to increase mental health literacy and communication between adolescents with lived experience with psychosis and their relatives.

**Disclosure of Interest:**

None Declared

